# Use of cannabidiol for off-label treatment of patients with refractory focal, genetic generalised and other epilepsies

**DOI:** 10.1186/s42466-025-00408-w

**Published:** 2025-07-22

**Authors:** Marie Hollander, Thomas Mayer, Kerstin Alexandra Klotz, Susanne Knake, Felix von Podewils, Gerhard Kurlemann, Ilka Immisch, Felix Rosenow, Susanne Schubert-Bast, Adam Strzelczyk

**Affiliations:** 1 Epilepsy Center Frankfurt Rhine-Main, Department of Neurology, Goethe-University Frankfurt, University Medicine Frankfurt, Theodor-Stern-Kai 7, 60596 Frankfurt am Main, Germany; 2 Epilepsy Center Kleinwachau, Radeberg, Germany; 3 Department of Pediatric Neurology, University Hospital Bonn, Bonn, Germany; 4 Department of Neuropediatrics and Muscle Disorders, Center for Pediatrics, Medical Center, Faculty of Medicine, University of Freiburg, University of Freiburg, Freiburg i.Br, Germany; 5 Epilepsy Center Hessen and Department of Neurology, Philipps-University Marburg, Marburg (Lahn), Germany; 6 Department of Neurology, Epilepsy Center, University Hospital Greifswald, Greifswald, Germany; 7 St. Bonifatius Hospital, Lingen, Germany; 8 Department of Pediatrics, Pediatric Epileptology, Goethe-University Frankfurt, University Medicine Frankfurt, Frankfurt am Main, Germany

## Abstract

**Background:**

In randomized controlled trials, add-on cannabidiol (CBD) has been shown to reduce seizure frequency in patients with Lennox-Gastaut syndrome, Dravet syndrome and Tuberous sclerosis complex. Real-world studies provide insights into the drug’s profile in other off-label indications. This study evaluated factors predicting efficacy, retention, and tolerability of add-on CBD used for off-label treatment in clinical practice for patients with refractory focal-onset, genetic generalised, and other unclassified epilepsies.

**Methods:**

A retrospective cohort study recruiting all patients who had started CBD between 2019 and 2023 for off-label treatment at six German epilepsy centres. Data on baseline and follow-up were obtained from patients’ medical records.

**Results:**

A total of 108 patients (mean age 27.3; median 36; range 1.4–68 years, 56 male) were treated with CBD. At three months, 42 (38.9% considering all 108 patients that started CBD) reported at least a 50% reduction in seizures, including 28 patients (25.9%) with a 50–74% reduction, and 14 (13%) with a reduction of 75–99%. Among those 48 patients experiencing tonic-clonic seizures (TCS), at least 50% response was reported by 45.8%, and eight (16.7%) patients were free of TCS. Sex, age, epilepsy syndrome, concomitant clobazam (CLB) use, and the number of concomitant or previous ASMs were not predictive of response. Mean seizure days per month significantly decreased from a mean of 16.8 (median: 13.5) to 14.5 (median 10, *p* = 0.002). The probability of patients remaining on CBD treatment was 85.2% (*n* = 92/108, 16 discontinuations) at three months, 73.5% at six months and 61.1% at twelve months; retention was better in children or adolescents compared to adults (log-rank *p* = 0.014). Using the CGI-C for overall impression, 69 (63.0%) patients were rated as very much, much, or minimally improved; for behaviour, 60 (55.6%) reported within this range of improvement. TEAEs were reported in 41 (38%) patients. The most frequent were diarrhoea (*n* = 15), sedation (*n* = 13), and nausea and vomiting (*n* = 7).

**Conclusions:**

Our results suggest CBD to be an effective ASM, with 50% responder rates similar to those observed in regulatory trials for other ASMs licensed in focal epilepsies. Its off-label use in refractory focal-onset, genetic generalised, and other unclassified epilepsies seems to be safe and well-tolerated.

## Highlights

Off-label use of cannabidiol in 108 patients with refractory focal, genetic generalised and other epilepsies.

Retention rate was 85.2% at three months, 73.5% at six months and 61.1% at 12 months follow-up.

50% responder rate was 39% at three months and 42% at one year.

Using CGI-C, 63.0% were rated as improved regarding overall impression and 55.6% regarding behaviour.

Adverse events were observed in 38% of patients, predominantly gastrointestinal symptoms (diarrhoea, nausea and vomiting) and sedation.

## Introduction

Anti-seizure medications (ASMs) play a central and crucial role in the treatment of people with epilepsy, the majority of whom require ASM treatment for an extended period of time. Since up to 30% of patients are refractory to ASM [1, 2], the development of new therapeutic options is strongly warranted. Due to ongoing seizures, patients with drug-refractory epilepsies are affected by increased risk of injury, morbidity and mortality, social stigma, reduced employment opportunities and impaired quality of life for both themselves and their caregivers [3–8]. Any newly introduced ASM would provide an opportunity to achieve better seizure control for some patients [9–11].

Cannabidiol (CBD, Epidyolex^®^) is an ASM recently approved by the European Medicines Agency (EMA) as an adjunctive therapy in patients aged ≥ 2 years of age for seizures associated with Lennox-Gastaut-Syndrome (LGS) or Dravet Syndrome (DS) in conjunction with clobazam (CLB), or as adjunctive therapy for seizures associated with Tuberous Sclerosis Complex (TSC). In the US, it is approved by the Food and Drug Administration (FDA) for the treatment of seizures associated with LGS, DS, or TSC in patients ≥ 1 year of age. These approvals were based on several double-blind, randomised controlled trials (RCTs) that showed the efficacy of CBD as an adjunctive therapy to standard ASMs in reducing drop seizures in LGS [12, 13], convulsive seizures in DS [14, 15], and TSC-associated seizures [16, 17] compared with placebo.

However, results from regulatory, clinical trials are difficult to extrapolate to clinical practice, as these studies are limited by their short duration, rigid inclusion and exclusion criteria, which exclude the majority of epilepsy patients, and lack of dosing flexibility [18, 19]. Upon the introduction of a new ASM, there is very limited information about the potential efficacy and tolerability in a naturalistic clinical setting, especially if, like CBD, they were tested in an orphan drug designation. In the absence of RCTs for off-label uses of CBD, real-world evidence offers essential clinical insights into its efficacy, tolerability and retention—a robust factor which combines aspects of both the former—across broader patient populations with epilepsy [20–23].

Our multicentre study aimed to give insights into retention, efficacy and tolerability in a large cohort of patients with different epilepsy syndromes during the first year of treatment with CBD. Furthermore, we aimed to identify predictors of efficacy and tolerability, and examined outcomes and treatment-emergent adverse events (TEAEs).

## Methods

### Study settings and design

This study was performed at six German epilepsy centres (Frankfurt am Main, Freiburg i. Br., Greifswald, Lingen [Münster], Marburg, Radeberg). All patients with refractory focal-onset, genetic generalised, and other unclassified epilepsies treated with CBD in one of the enrolling epilepsy centres between 2019 and 2023 were included. The retrospective analysis was approved by the ethics committee of the University of Frankfurt. Informed consent was waived due to the retrospective nature of the study design, and STROBE (Strengthening the Reporting of Observational Studies in Epidemiology) guidelines were followed [24]. The study was not sponsored or funded by any third party.

The epilepsy diagnosis was based on the definitions proposed by the ILAE and the International Bureau for Epilepsy [25, 26]. No patients treated on-label for LGS [27], DS [28] or TSC [29] were included in the present analysis; these analyses are reported separately [30]. The treating physician at each study site provided information on epilepsy syndrome, aetiology, semiology, demographics, concomitant and previous ASMs, and seizure frequency in the three months prior to CBD treatment (defined as baseline). Patients were interviewed about the occurrence of TEAEs at each visit, and TEAEs were documented according to WHO criteria. Patients were usually seen every three to six months. Follow-up data, collected through patients’ medical records, included target and maximal doses of CBD, seizure frequency, TEAEs, retention of CBD, and discontinuation of CBD categorised according to the following reasons: TEAEs, lack of effectiveness, both TEAEs and lack of effectiveness, or not reported.

Seizure reduction was analysed at three-, six- and 12-month follow-ups regarding the preceding 3, 6, 12 months period since start of CBD for total seizures and tonic-clonic seizures (TCS, this term includes focal to bilateral tonic-clonic, bilateral tonic-clonic and generalised tonic-clonic seizures according to the updated ILAE criteria [31]), where data was available. A 25% responder rate was defined as a 25% or greater seizure reduction compared to the defined baseline, a 50% responder rate meant a 50% or greater seizure reduction, and a 75% responder rate meant a 75% or greater seizure reduction compared to baseline. No response was defined as a change (decrease or increase) in seizure frequency by less than 25% compared to baseline. Seizure increase was defined as a 25% or greater increase in seizure frequency compared to baseline. In addition, change in seizure occurrence was recorded as the average number of seizure days per month, regardless of seizure type at baseline and final follow-up. Physicians rated the overall and behavioural clinical change (CGI-C) during CBD treatment on a 7-point rating scale, categorised from very much improved to very much worse. Retention was defined as patients continuing CBD treatment after three, six and 12 months, and the rate was estimated using Kaplan-Meier survival curves.

### Data entry and statistical analysis

The statistical analyses described above, including descriptive analyses, were performed using IBM SPSS Statistics, version 28 (IBM Corp., Armonk, NY, USA). Retention time was displayed using Kaplan-Meier survival curves. Mann-Whitney-U, chi-squared and log-rank tests were used for statistical analysis, and *p*-values < 0.05 were regarded as statistically significant.

## Results

### Patient characteristics at baseline

A total of 108 patients with a mean age of 27.3 ± 14.7 years (median 26; range 1.4–68 years) were treated with CBD, of whom 56 patients were male (51.9%). Among the cohort were 32 children and adolescents (29.6%), with 76 (70.4%) adults 18 years or older. The patients had a mean epilepsy duration of 17.8 ± 11.7 years (median 16; range 1–57 years) with a mean age of onset of 8.8 ± 9.6 years (median 5.0; range 0.1–44.0 years). All patients had drug-refractory epilepsy; 74 (68.5%) had focal-onset epilepsy, 14 (13.0%) had genetic generalised epilepsy, and 20 (18.5%) had other or unclassified epilepsy. They were taking a mean of 2.7 ± 1.0 ASMs (median: 3, range: 1–6 ASMs) before starting CBD. Concomitant use of CLB was reported in 44 (40.7%) patients, with a mean dose of 10.7 mg (median 10, range 2.5–30 mg) corresponding to a mean of 0.16 mg per kg bodyweight (median 0.13; range 0.03–0.42). Other mainly used ASMs were lamotrigine (*n* = 37, 34.3%, mean dose 363 mg), valproate (*n* = 36, 33.3%, mean dose 1318 mg), brivaracetam (*n* = 32, 29,6%, mean dose 246 mg), perampanel (*n* = 28, 25.9%, mean dose 8 mg), lacosamide (*n* = 27, 25%, mean dose 400 mg), and topiramate (*n* = 19, 17.6%, mean dose 199 mg). In the past, the patients had experienced failed treatment with a mean of 6.8 ± 3.6 ASMs (median: 7, range: 1–18 failed ASMs; current ASM not included).

### Treatment with Cannabidiol

The starting dose of CBD in patients varied between 12.5 mg and 1200 mg per day, with a mean of 170.1 ± 155.6 mg and a median of 100 mg (median 2.2 mg per kg bodyweight per day, 2.8 in children vs. 1.8 in adults, *p* = 0.017). The target dose ranged between 100 mg and 1600 mg, with a mean of 628.8 ± 333.3 mg and a median of 600 mg (median 10 mg per kg bodyweight per day, 16.7 in children vs. 9.1 in adults, *p* < 0.001); target dose was achieved within a median time of 3 weeks. The maximum dose ranged between 100 mg and 2400 mg with a mean of 791.5 ± 468.8 mg and a median of 700 mg (median 12.8 mg per kg bodyweight, 22.7 in children vs. 10.9 in adults, *p* < 0.001).

### Outcome and predictors of response at three months

**Fig. 1:**
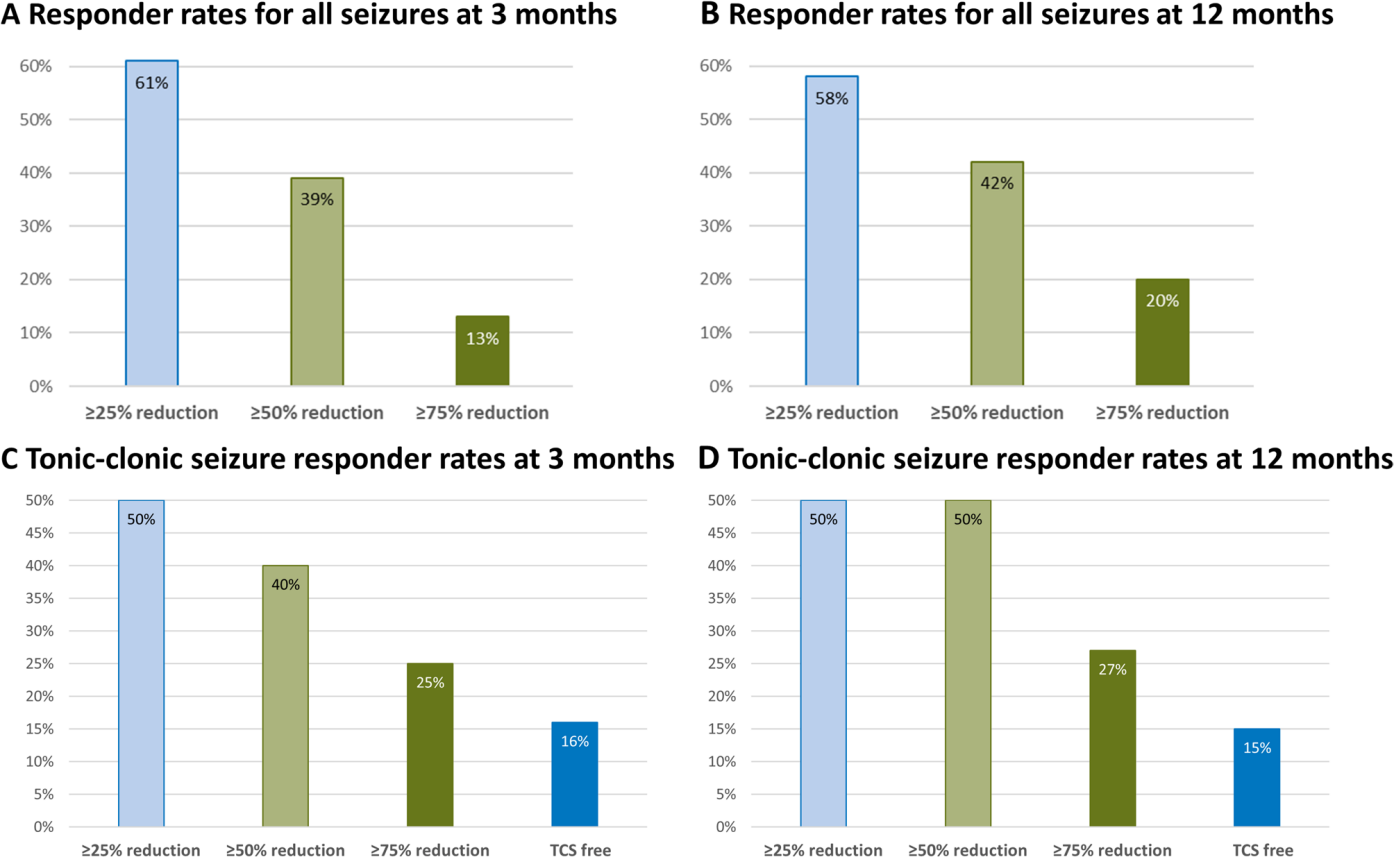
Responder rates at three months for (**A**) total seizures and (**B**) tonic-clonic seizures (TCS), and at 12 months for (**C**) total seizures and (**D**) tonic-clonic seizures (TCS)

At three months of follow-up, 66 (61.1%, 66/108 considering all patients that started CBD) reported a 25% or greater reduction in seizures, there was no difference between those on (*n* = 27/44; 61.4%) and without CLB (*n* = 39/64; 60.9%; *p* = 0.964). This included 24 patients (22.2%) with a 25–49% reduction in seizures, 28 patients (25.9%) with a 50–74% reduction, and 14 (13%) with a reduction of 75–99%. Responder rates are presented in Fig. 1A. In 31 patients (28.7%), the seizure frequency was unchanged, while six patients (5.6%) reported an increase. For five patients, no seizure outcome data was available.

Of the 48 patients experiencing TCS, five (10.4%) reported a 25–49% reduction of TCS, seven (14.6%) reported a 50–75% reduction, and four (8.3%) reported a 75–99% reduction. Eight (16.7%) patients reported being free of TCS. There was no difference in TCS response between those on and without CLB (*p* = 0.768). Two (4.2%) patients had an increase in TCS, while in six (12.5%), TCS frequency was unchanged; for details, please refer to Fig. 1C. Data was unavailable for 16 patients. Table 1 shows details for responders and non-responders in terms of sex, age, epilepsy syndrome, concomitant CLB use, concomitant and previous number of ASMs. No significant differences were found in the chi-squared analysis (*p* > 0.05 across all parameters).

**Table 1:** Clinical characteristics and outcome on follow-up of 3 months

	all patients *n* = 108	non responders *n* = 42	responders *n* = 66	*p*-value
	*n*	% (*n*)		
**total**	108	38.9 (42)	61.1 (66)	
**sex**				0.758
male	56	37.5 (21)	62.5 (35)	
female	52	40.4 (21)	59.6 (31)	
**age range**				0.055
< 18 years	32	25 (8)	75 (24)	
≥ 18 years	76	44.7 (34)	55.3 (42)	
**Epilepsy syndrome**				0.925^#^
Focal-onset epilepsy	74	39.2 (29)	60.8 (45)	
Genetic generalised epilepsy	14	50 (7)	50 (7)	
other and unclassified	20	30 (6)	70 (14)	
**Concomitant use of clobazam**				0.964
Use of clobazam	44	38.6 (17)	61.4 (27)	
No use of clobazam	64	39.1 (25)	60.9 (39)	
**number of concomitant ASM at start of CBD**				0.203
1–2 ASM	52	32.7 (17)	67.3 (35)	
3 or more ASM	56	44.6 (25)	55.5 (31)	
**previously failed ASMs (without current)**				0.820
6 ASM and below	27	37.0 (10)	63.0 (17)	
≥ 7 ASM	81	39.5 (32)	60.5 (49)	

ASM, anti-seizure medication; CBD, cannabidiol; ^#^focal vs. non-focal

### Seizure days

At baseline, patients reported a high seizure burden, with 16.8 ± 11.3 seizure days per month (median: 13.5, range: 0–30). The burden was significantly higher in children and adolescents, with 22.1 ± 11 seizure days per month (median: 30, range: 1–30), than in adults, who had 14.7 ± 10.8 seizure days per month (median: 12, range: 0–30, *p* = 0.011) during the three-month baseline phase. One adult had been seizure-free during the three months of baseline prior to treatment.

**Fig. 2:**
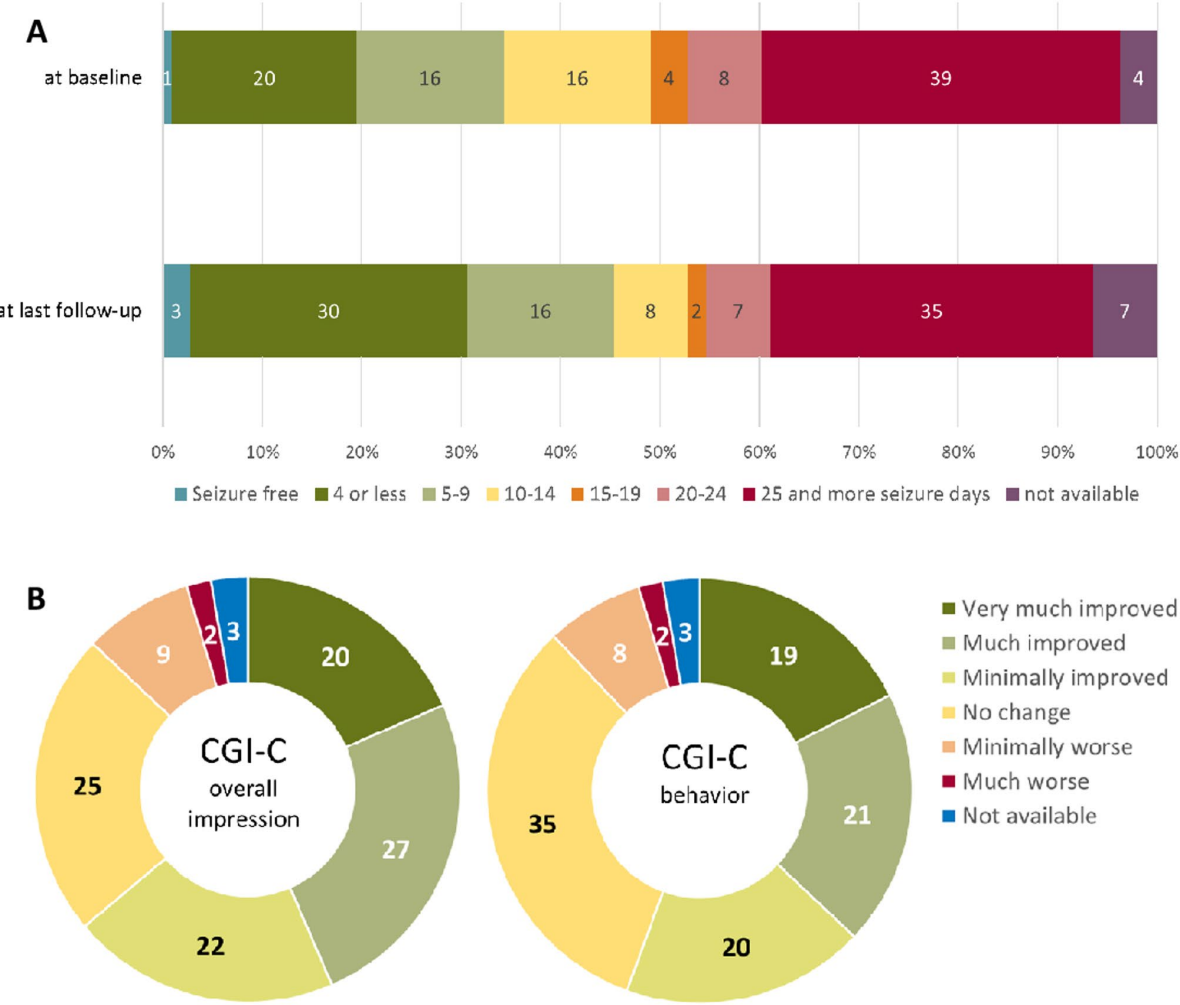
(**A**) Percentage of patients according to seizure days per month across seven incremental categories at baseline and final follow-up, after initiation of cannabidiol (**B**) Physician-assessed Clinical Global Impression of Change (CGI-C) for the overall impression and (**C**) behavioural change

At the final follow-up point, the mean seizure days per month significantly decreased to 14.5 (median 10, range 0–30) days per month in the previous three months of CBD treatment (*p* = 0.002). Among children and adolescents, seizure days reduced to a mean of 20.2 (median 27.5, *p* = 0.064), and among adults to 12.2 (median 7.5, *p* = 0.011) seizure days per month. Figure 2A shows the number of seizure days per month at baseline and final follow-up.

### Retention analysis and long-term response

**Fig. 3:**
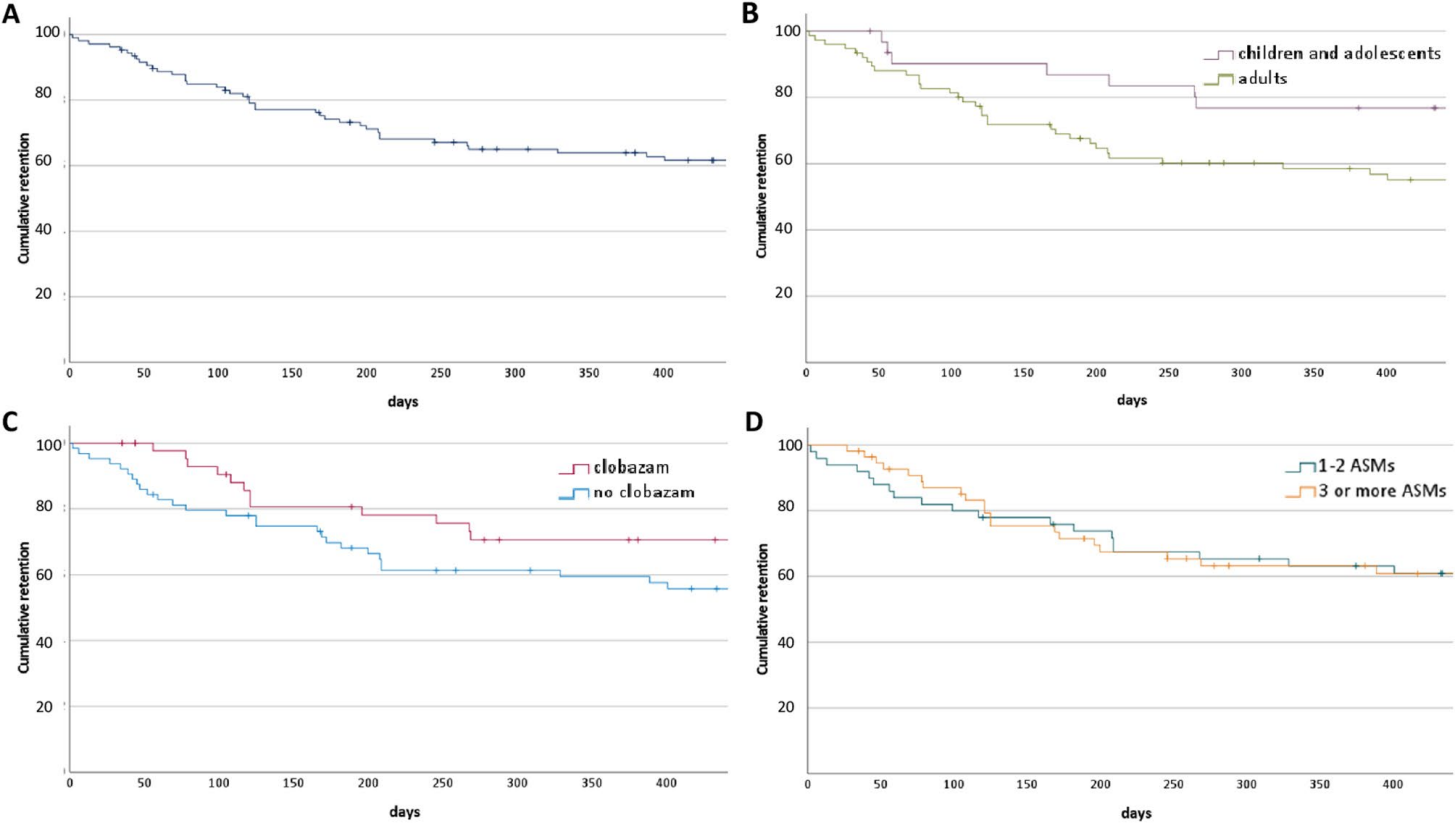
Retention rate of cannabidiol (CBD) in the complete cohort (**A**), and stratified by age (**B**; long-rank *p*-value: 0.014); concomitant clobazam (**C**; long-rank *p*-value: 0.062), for number of concomitant ASMs (**D**; long-rank *p*-value: 0.699), ASM = anti-seizure medication

The probability of patients remaining on CBD treatment was 85.2% (*n* = 92/108, 16 discontinued) at three months, 73.5% (*n* = 75/102, 27 discontinued, 6 with no-follow-up) at six months and 61.1% (*n* = 58/95, 37 discontinued, 13 with no-follow-up) at twelve months. Kaplan-Meier survival curves show the retention over time (Fig. 3A). The retention was better in children and adolescents compared to adults (log-rank *p* = 0.014, Fig. 3B). There was no difference observed regarding comedication with CLB (log-rank *p* = 0.062, Fig. 3C) or the number of concomitant ASMs (log-rank *p* = 0.699, Fig. 3D).

The reasons for discontinuation of CBD at one year were TEAEs (*n* = 16, 14.8%), insufficient efficacy (*n* = 16, 14.8%), or both (*n* = 5, 4.6%). TEAEs associated with discontinuation were mainly diarrhoea (*n* = 7; 6.5%), nausea and vomiting (*n* = 6, 5.6%), and sedation (*n* = 5; 4.6%). For details, please refer to Table 2.

**Table 2:** Characteristics of reported treatment-emergent adverse events on cannabidiol, their frequency and related discontinuation at 12 months (total *n* = 108)

Adverse events	Reported *n* (%)	Leading to withdrawal *n* (%)
**Overall**	41 (38)	21 (19.5)
**GI symptoms**	21 (19.5)	11 (10.2)
Diarrhoea	15 (13.9)	7 (6.5)
Nausea / vomiting	7 (6.5)	6 (5.6)
Loss of appetite / weight loss	3 (2.8)	1 (< 1)
**Sedation**	**13 (12.0)**	**5 (4.6)**
**CNS/Ataxia**	3 (2.8)	2 (1.9)
Dizziness	2 (1.9)	1 (< 1)
Ataxia	1 (< 1)	1 (< 1)
**Psychobehavioural**	**10 (9.3)**	**7 (6.5)**
Aggression / Irritability	3 (2.8)	2 (1.9)
Behavioural	3 (2.8)	2 (1.9)
Anxiety	1 (< 1)	1 (< 1)
other	3 (2.8)	2 (1.9)
**Skin**	**3 (2.8)**	**1 (< 1)**
**Elevated liver enzymes**	**1 (< 1)**	**1 (< 1)**
Other	3 (2.8)	1 (< 1)

Among those on treatment at 12 months, 41 reported a 25% or greater reduction in seizures (57.7%, 41/71). This included 11 patients (15.5%) with a 25–49% reduction, 16 patients (22.6%) with a 50–74% reduction and 14 patients (19.7%) with a 75–99% reduction in seizure frequency; details are presented in Fig. 1B. In patients experiencing TCS (*n* = 26) at 12 months, 13 (50%) reported a 50% or greater reduction. Among these, four (15.4%) patients were free of TCS, six (23.1%) had a 50–75% reduction, and three (11.5%) had a 75–99% reduction. In three patients, TCS frequency was unchanged, and for ten patients, no data was available. Details are presented in Fig. 1D.

### Overall change, behavioural change and treatment-emergent adverse effects

Using the CGI-C for overall impression, 20 (18.5%) patients were rated as very much improved at the last follow-up, 27 (25%) patients were much improved, and 22 (20.4%) patients were minimally improved. Twenty-five (23.1%) patients showed no change. Nine (8.3%) patients were rated minimally worse and two (1.9%) were rated much worse. No patients were rated as very much worse (Fig. 2B). The CGI-C for behaviour showed 19 (17.6%) patients rated as very much improved, 21 (19.5%) as much improved, and 20 (18.5%) as minimally improved. For further details, please refer to Fig. 2C.

Treatment-emergent adverse effects (TEAEs) were reported in 41 (38%) patients. The most frequent were diarrhoea (15 patients), sedation (13 patients), and nausea and vomiting (7 patients). For details, please refer to Table 2.

## Discussion

This study reflects experiences with off-label use of CBD during the first years of market access in a cohort of 108 patients with drug-refractory focal-onset, genetic generalised and other epilepsy syndromes, excluding LGS, DS or TSC.

The observed efficacy of CBD was well in line with that observed during RCTs and open-label extension studies for LGS [12, 13], DS [14, 15], and TSC [16], with 50% responder rates of 39% at three months and 42% at one year. The retention rates at three (85%), six (73%), and 12 months (61%) are encouraging, and align well with findings from other post-marketing studies on CBD [32–34]. In addition, the retention is similar to or above that of other ASMs reported in post-marketing studies, including brivaracetam, cenobamate, eslicarbazepine, lacosamide, lamotrigine, levetiracetam, perampanel, topiramate, valproate, and zonisamide in focal or generalised epilepsies [35–47], however, any direct comparisons should be interpreted with caution. In particular, retention data may appear favorable; but cross-study comparisons are inherently limited due to differences in study design and the lack of head-to-head trials [48].

So far, data on the use of CBD in genetic generalised epilepsies has been heterogeneous [49, 50]; however, in our study, patients with this epilepsy type exhibited a 50% responder rate of 52.6% (10/19), suggesting a role for CBD in managing some of these patients. Our results suggest that CBD may offer clinical benefits in certain cases of treatment-resistant epilepsies beyond currently approved indications [51]; however, such use should be considered exploratory, and further prospective studies are needed to establish its efficacy and safety across different epilepsy syndromes.

Mechanism-of-action data support this observation, suggesting that CBD may achieve anti-seizure effects through various pathways, including the modulation of intracellular calcium via GPR55 and TRPV1 receptors and the regulation of adenosine signalling [52]. Although the exact mechanisms remain under investigation, these targets appear to act broadly rather than being limited to specific epilepsy aetiologies or syndromes [52].

Findings on differences in retention rates of CBD between children and adults differ across studies. We observed better retention and higher median daily dosage per kg of body weight for CBD in children, with 22.7 mg per kg compared to 10.9 mg per kg body weight in adults. Higher weight-adjusted dosing in children was also reported in earlier studies on the use of CBD [32, 53]. A retrospective, single-centre study from the University of Wisconsin–Madison [54] reported no differences in retention rates, with 77.2% of paediatric patients (*n* = 57) and 76.5% of adult patients (*n* = 51) remaining on CBD over an average of 20 months. In contrast, results from the Italian Expanded Access Program showed higher retention rates in adults than children [55]. Expanded Access Program data from the US revealed no significant differences in seizure frequency reduction between children up to 2 years and adults [56].

The lower-than-recommended median starting dose of 2.2 mg per kg bodyweight per day reflects cautious titration practices in clinical routine, particularly in patients with complex therapy with multiple ASMs or concern for drug interactions. This conservative approach is commonly adopted to improve tolerability, especially in populations outside of the licensed indications. Overall, the TEAE profile observed in our study was in line with the established adverse events profile of CBD. Overall, TEAEs were reported in 37.9% of patients, with discontinuation due to TEAEs occurring in 19.5% of the cohort. The most common TEAEs were gastrointestinal symptoms and sedation. We did not observe any serious TEAEs, and, similar to other post-marketing studies, the overall proportion of patients reporting TEAEs was lower than in RCTs where TEAEs are more rigorously documented. Systematic literature reviews of clinical trials have indicated that CBD is associated with somnolence (particularly when used with CLB), as well as decreased appetite, diarrhoea, and elevated liver enzymes, especially in conjunction with valproate [57–59]. We observed elevated liver enzymes in only one patient; however, data on laboratory tests were not routinely collected. Patients with epilepsy are prone to behavioural and psychiatric TEAEs as well as sleep disorders, with some ASMs reported to worsen these symptoms [60]. In the current study, consistent with findings from other CBD studies, psychobehavioural TEAEs were infrequently observed. In addition, physicians rated 55.6% of patients as showing improvement or no change (32.4%) in behaviour. Overall, CBD appears to have a favourable psychobehavioural profile.

This study is subject to several limitations typical of retrospective chart reviews, including an uncontrolled design, potential gaps or incompleteness in the data, and variability in data collection processes. For example, the absence of detailed records on total seizure counts and TCS may have influenced the accuracy of responder rate calculations. Moreover, seizure frequency data were based on caregiver-reported seizure diaries, which depend on their reliability and accuracy, and may be subject to over- and underreporting [61]. Therapeutic drug monitoring (TDM) was not routinely performed or collected for CBD and other concomitant ASM. Implementing routine TDM for CBD and co-administered ASMs could help minimize TEAEs and support informed dosing adjustments. Recent progress was reported in TDM techniques for precise CBD measurement, that offers significant utility in assessing drug-drug interactions, and might contribute to optimized patient care in complex therapeutic scenarios [62]. The study also lacked a predefined design to statistically evaluate differences between age subgroups, epilepsy types, or the impact of concomitant CLB use. These limitations highlight the need for prospective, controlled studies to provide a more comprehensive understanding of the findings.

## Conclusions

In conclusion, our analysis demonstrates that CBD can achieve favourable responder rates in clinical practice, along with good tolerability, among patients with drug-refractory focal-onset epilepsy, genetic generalised epilepsy, and other epilepsy syndromes.

## Data Availability

The data of this study are available from the corresponding author upon reasonable request.
